# Supplementary material for: A non-canonical sensing pathway mediates Plasmodium adaptation to amino acid deficiency
Source: Commun Biol. 2023 Feb 21;6:205. doi: 10.1038/s42003-023-04566-y (PMC9942083; doi:10.1038/s42003-023-04566-y)
Supplement: Supplementary file 2 — Supplementary Information [file 42003_2023_4566_MOESM2_ESM.pdf]

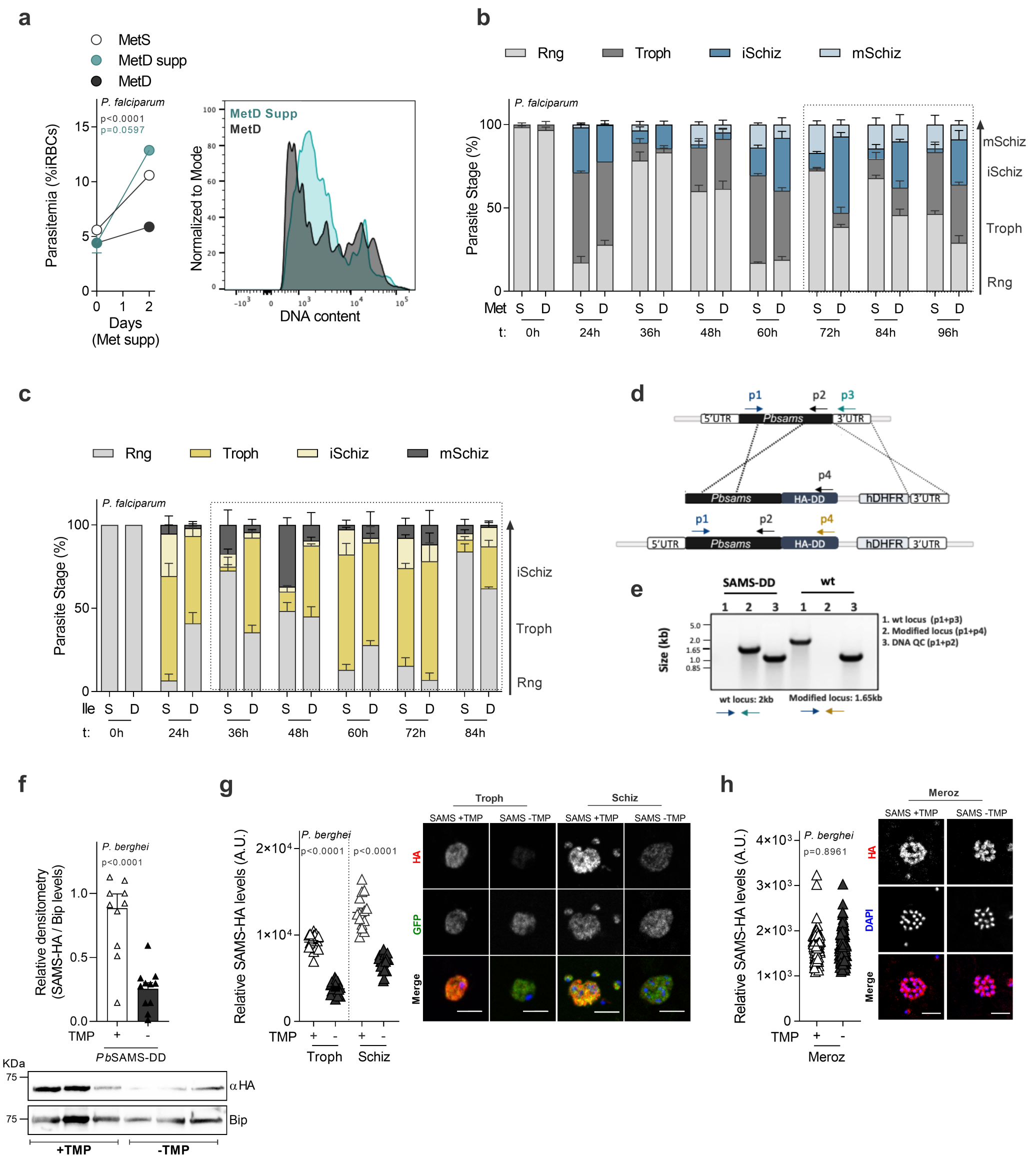

Supplementary Figure 1 - AA depletion impacts parasite intra-erythrocytic development but not viability. (a) Parasitemia of synchronized *P. falciparum* WT parasites cultured in MetS, MetD or MetD media supplemented with methionine (MetD supp). Methionine was provided to the media at the concentration found in RMPI (100µM) from day 4 to day 6 after sorbitol synchronization. Parasitemia was measured by flow cytometry after SYBR Green staining. **(b, c)** Full course analysis of *Pf* 3D7 WT developmental stage progression in **(b)** MetS or MetD media and **(c)** IleS or IleD media for 2 developmental cycles. Parasite staging was performed by microscopy analysis of Giemsa-stained blood smears. Individual data points, for each timepoint and condition, are provided in **Supplementary Figure 2**. **(d)** Double crossover homologous recombination at the *Pbsams* locus and the 3'UTR was employed to introduce a fusion of *Pbsams* ORF with the destabilizing domain system (DD) and an HA tag (*Pb*SAMS-DD line). Annealing sites for genotyping primers are illustrated and primer sequences are given in **Supplementary Table 1**. **(e)** Agarose gel image, showing diagnostic PCR products from *P. berghei* SAMS-DD and *P. berghei* WT genomic DNA, after dilution cloning

of the *Pb*SAMS-DD transgenic parasite line. **(f)** Densitometry analysis of SAMS-HA-DD protein levels in BALB/c mice infected by intraperitoneal inoculation of $1x10^6$ *Pb*SAMS-DD infected RBCs (iRBCs). SAMS protein levels were probed for the HA-tag (*Pb*SAMS-HA-DD line; αHA, 70KDa). Bip was employed as loading control. **(g, h)** Representative image and quantification of SAMS protein levels at the **(g)** trophozoite (Troph), schizont (Schiz) and **(h)** merozoite (Meroz) stages in *Pb*SAMS-DD parasites developing in BALB/c mice treated or not with TMP, measured by fluorescence microscopy after probing for the HA-tag. **a.** Data represents the mean percentage of iRBCs in n=2 independent experiments, each performed at least in triplicate. **b, c.** Data is shown as mean percentage of parasites at each developmental stage with error bars representing SEM. n=3 independent experiments. The dashed squares represent the temporal window during which amino acid deprivation has the greatest impact on parasite development. **f.** Representative western blot image, showing SAMS stabilization after TMP treatment. Data is shown as mean ± SEM of SAMS-HA-DD levels normalized to Bip levels (Mann-Whitney). Data represents n=3 independent experiments and each lane represents an individual mouse (10 mice per condition). **g, h.** Scatter dot plots represent SAMS intensity levels in n=2 independent experiments (2 mice /condition). Data is shown as SAMS mean intensity values inside *Pb*SAMS-DD +TMP and *Pb*SAMS-DD -TMP parasites, normalized to parasite area (Mann-Whitney). Scatter dot plots show the data for the following number of parasites (N): Trophozoite-stage: SAMS-DD: +TMP, N=20; -TMP, N=20; Schizont-stage: SAMS-DD: +TMP, N=15; -TMP, N=21 and Merozoite-stage: SAMS-DD: +TMP, N=102; -TMP N=108. Scale bars = 5μm.

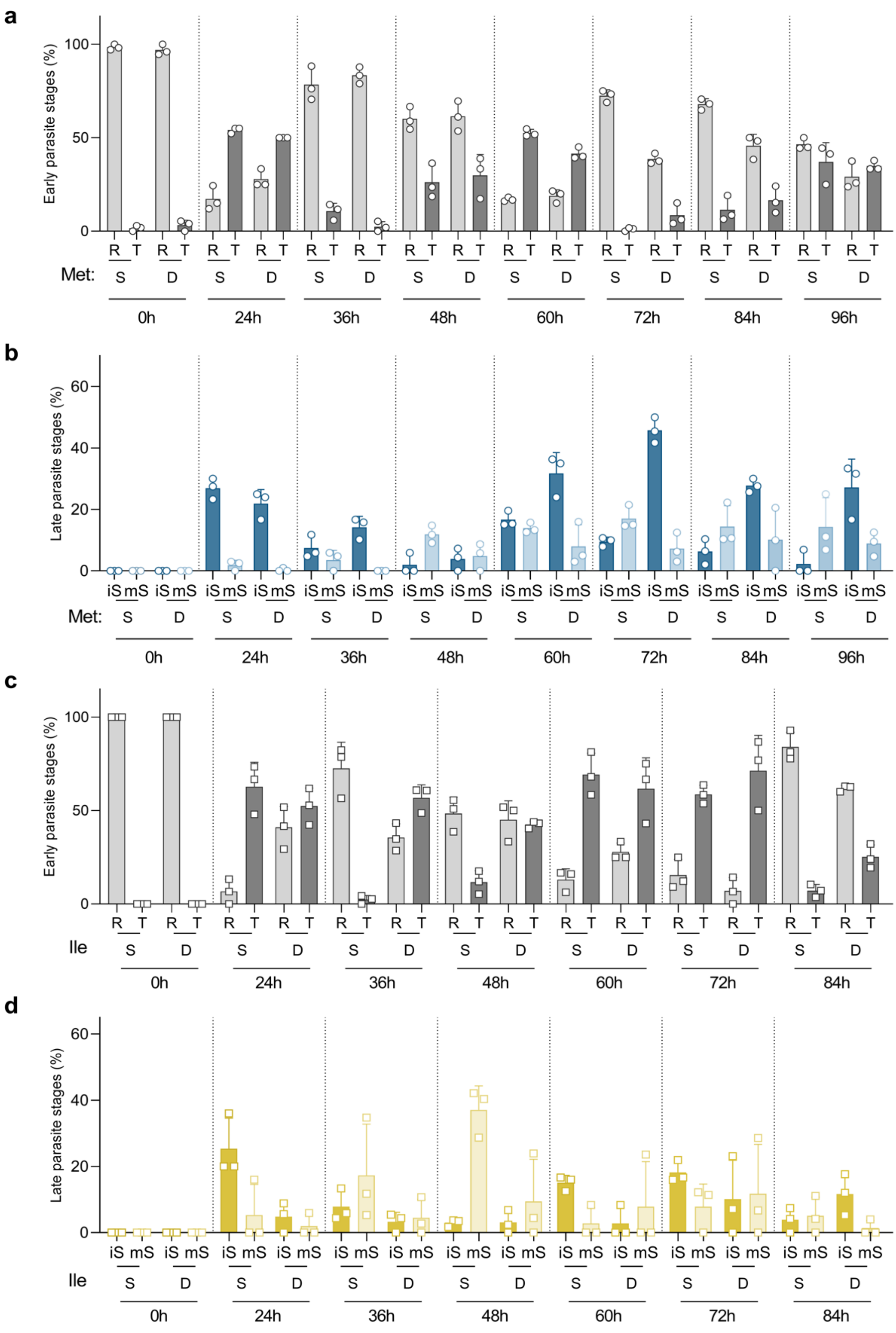

Supplementary Figure 2- Impact of AA depletion throughout *P. falciparum* intra-erythrocytic development. (a,b) Quantification of **(a)** ring- and trophozoite- parasite stages (early parasite stages) or **(b)** immature- and mature- schizont stages (late parasite stages) in MetS or MetD media. **(c,d)** Quantification of **(c)** ring- and trophozoite- stages or **(d)** immature- and mature- schizont stages in IleS or IleD media. Data represents the mean percentage of parasites at each developmental stage with individual datapoints representing independent experiments (*n*=3). Error bars represent SD. R= ring; T= trophozoite; iS = immature schizont; mS= mature schizont.

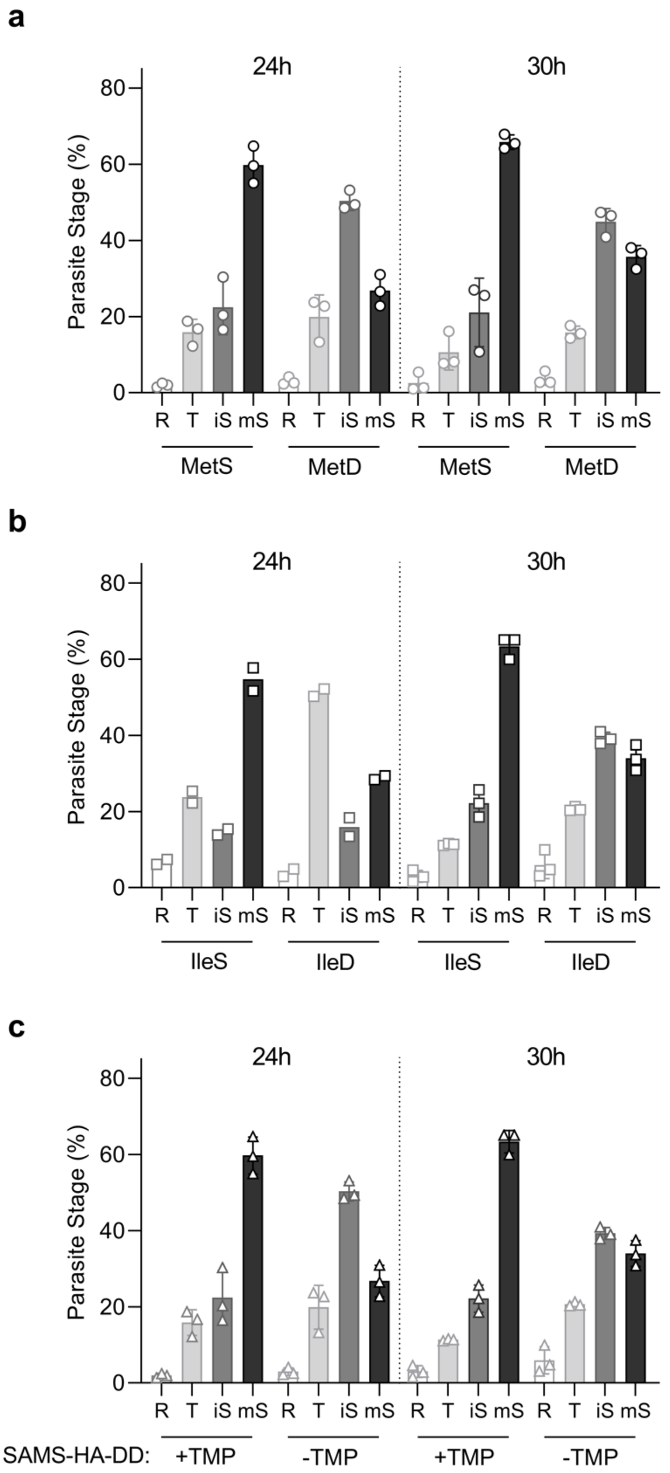

Supplementary Figure 3 – Impact of AA depletion in *P. berghei* intra-erythrocytic development using the *ex vivo* maturation assay. (a-c) Quantification of *P. berghei* developmental stage progression under **(a)** MetS and MetD or (**b)** IleS and IleD media and in **(c)** SAMS knockdown parasites when cultured *ex vivo* for 24 or 30 hours. Data represents the mean percentage of parasites at each developmental stage with individual datapoints representing independent experiments. *n*=3 independent experiments, except for IleS, IleD at 24h (*n*=2). Error bars represent SD. R= ring; T= trophozoite; iS = immature schizont; mS= mature schizont.

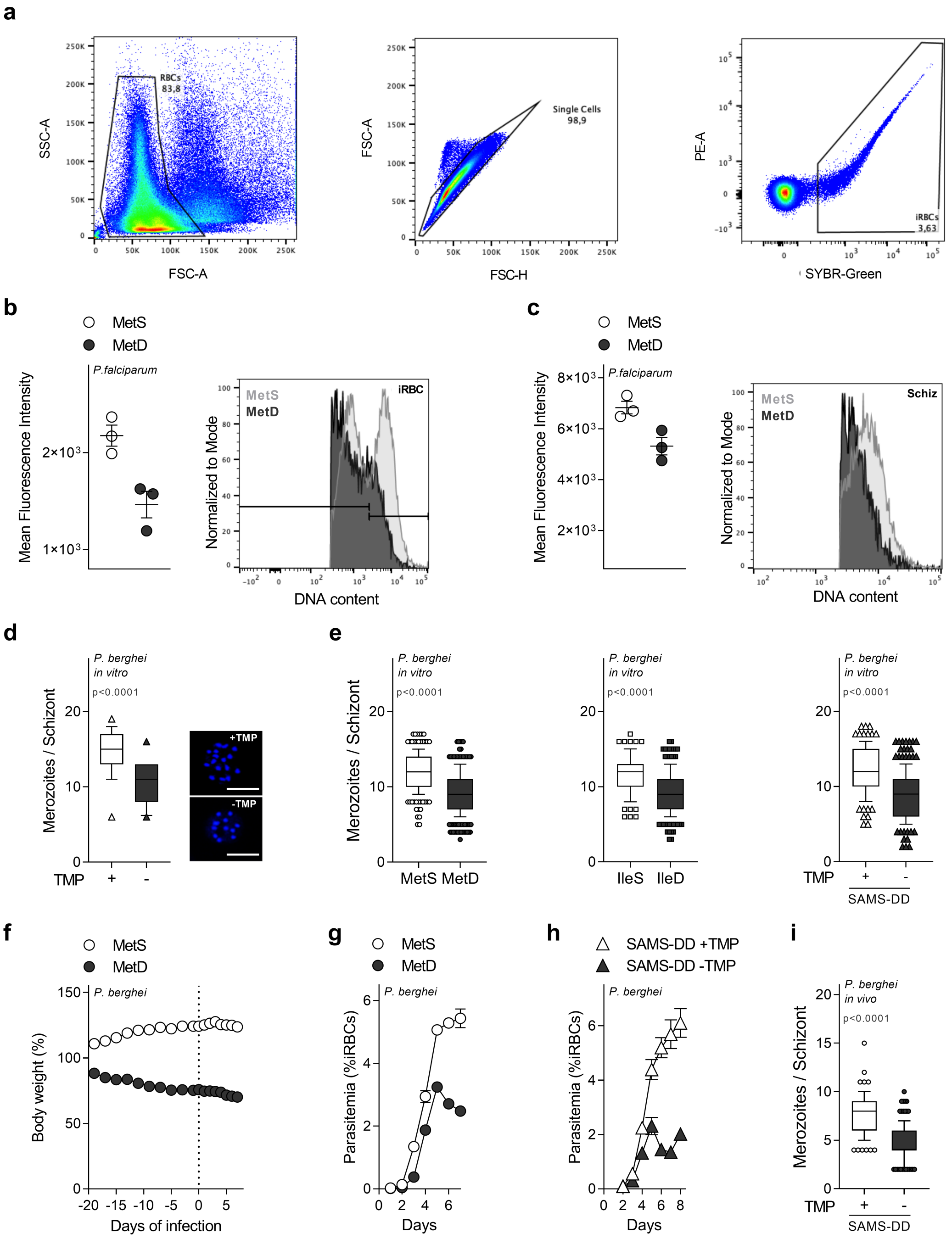

Supplementary Figure 4 – Effect of AA depletion *in vitro*, *ex vivo* and *in vivo*. (a) Representative image of flow cytometry plots and gating strategy for analysis of synchronized *P. falciparum* parasites cultured in MetS or MetD media after SYBR-Green staining. Samples were analyzed on BD LSRFortessa and the number of total acquired events ranged from 50 000 to 10 000/condition. The data was further processed on FlowJo™ 10 Software (Tree Star Inc.). Single RBCs were selected on the basis of their size (left and middle panel) and, subsequently, on the DNA content, excluding false positives associated to RBC autofluorescence (right panel)**. (b, c)** Mean Fluorescence Intensity (MFI) in **(b)** RBCs infected with synchronized *Pf*NF54 WT parasites or **(c)** *Pf*NF54 WT schizonts cultured in MetS or MetD media**.** Histogram plot showing the fluorescent

intensity comparison between MetS and MetD. **(d)** Mean merozoite numbers in *P. berghei* SAMS-DD parasites, treated or not with TMP, using the *ex vivo* maturation assay. Mature schizonts were visualized by microscopy after DNA staining with DAPI and quantified using the ImageJ counter tools. Representative fluorescent microscopy image shows the mean merozoite number comparison between SAMS-DD +TMP and SAMS-DD -TMP (knockdown) parasites. Scale bar= 5µm. **(e)** Box plot of mean merozoites number per schizont of *P. berghei* parasites after *ex vivo* culture for 30h, in MetS- or MetD-media, IleS- or IIeD-media and in SAMS knockdown parasites. **(f)** Body weight change in BALB/c male mice under a methionine restriction-regimen (MetD) or in regular diet, sufficient for Met (MetS). **(g)** Parasitemia of BALB/c mice (15mice/group) under a MetS or MetD regimen, infected by *i.p.* injection of $1x10^6$ GFP-expressing *P. berghei* ANKA WT iRBCs. **(h)** Parasitemia of MetS-fed BALB/c mice infected with *Pb*SAMS-DD parasites and treated, or not, with TMP in drinking water. **(i)** Mean merozoite number per schizont of *P. berghei* SAMS-DD parasites developing *in vivo* in MetS-fed BALB/c mice treated or not with TMP. **b, c.** Data is shown as scatter dot plot and represents 1 of *n*= 2 independent experiments (3 mice/condition). **d, e.** Data is represented as box-whisker plot (10-90 percentile) of mean merozoite number per schizont ± SD (Mann-Whitney). Boxplots show the data of **d.** *n*=2 or **e.** *n*=3 independent experiments and for the following number of schizonts (*N*): **d.** MetS, *N*=20; MetD, *N*=20; **e.** MetS, *N*=261, MetD, *N*=340; IleS, *N*=113, IleD, *N*=192; SAMS-DD +TMP, *N*=163, SAMS-DD -TMP, *N*=200. **f.** Data is represented as mean percentage of body weight change. Body weight data was normalized to the initial weight of each animal and examined in *n*=2 independent experiments. **g, h.** Data is represented as mean percentage of iRBCs ± SEM, determined in *n*=2 (MetS, MetD) or *n*=3 (SAMS-DD-HA) independent experiments. **i.** Data is represented as box-whisker plot (10-90 percentile) of mean merozoite number per schizont ± SD (Mann-Whitney). Boxplots show the data of *n*=3 independent experiments and for the following number of schizonts (*N*)**:** SAMS-DD +TMP, *N*=152; SAMS-DD -TMP, *N*=131. Scale bars= 5 µm.

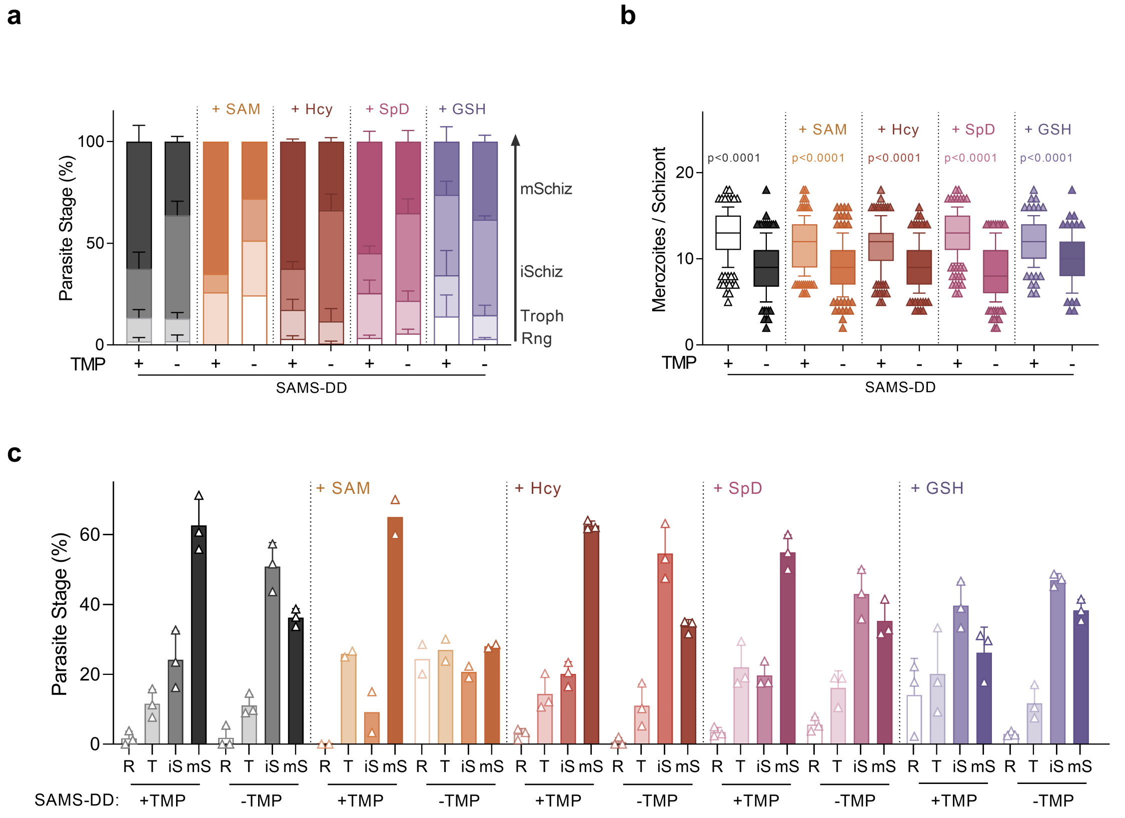

Supplementary Figure 5 – Effect of Met-downstream metabolites in *Plasmodium* development and replication. (a, b) Quantification of **(a)** intra-erythrocytic developmental stages and **(b)** mean merozoite numbers in *P. berghei* parasites knockdown for the SAMS enzyme after supplementation with S-adenosylmethionine (SAM), homocysteine (Hcy), spermidine (SpD) and glutathione (GSH). **(c).** Percentage of rings, trophozoites, immature and mature schizonts in *P. berghei* SAMS-DD knockdown parasites growing in media supplemented with the Met-downstream metabolites. **a.** Parasite staging was performed in triplicate, averaged and repeated in *n*=3 independent experiments, except for the SAM metabolite (*n*=2). Data is shown as mean percentage of parasites at each developmental stage, with error bars representing SEM. Individual data points are represented in **c.** Rng: ring; troph: trophozoite; iSchiz: immature schizont; mSchiz: mature schizont. **b.** Data is represented as box-whisker plot (10-90 percentile) of mean merozoite number per schizont ± SD (2-way ANOVA). Boxplots show the data of *n*=3 independent experiments, except for the SAM metabolite (*n*=2) and for the following number of schizonts (*N*): SAMS-DD: +TMP, *N*=200; -TMP, *N*=210; +TMP +SAM, *N*=141; -TMP +SAM, *N*=135; +TMP +Hcy, *N*=130; -TMP +Hcy, *N*=129; +TMP +GSH, *N*=102; -TMP +GSH, *N*=83; +TMP +SpD, *N*=154; -TMP +SpD *N*=154. **c.** Bars represent the mean percentage of parasites at each developmental stage with individual data points representing each independent experiment. *n*=3 independent experiments, except for the SAM metabolite (*n*=2). Error bars represent the SD.

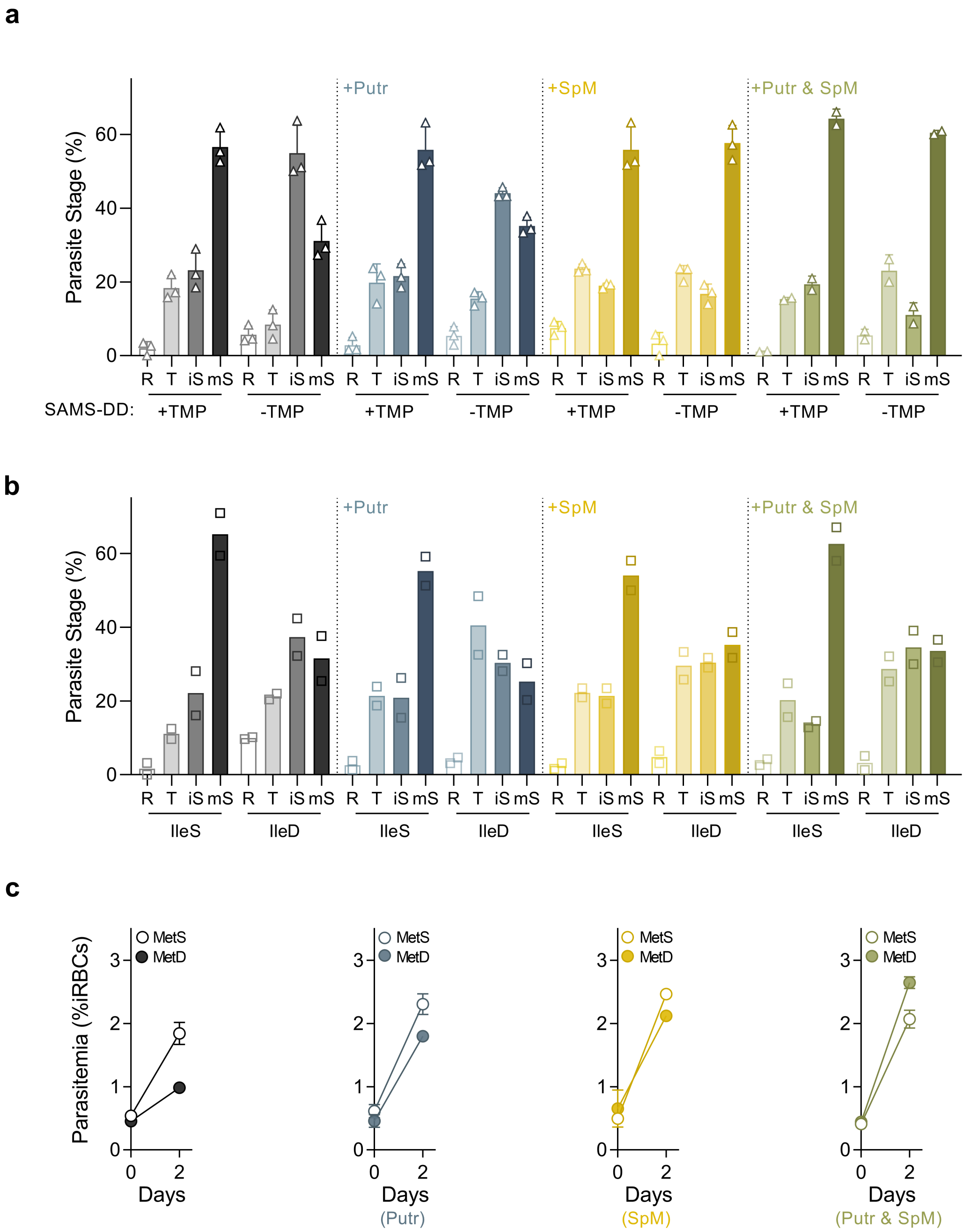

Supplementary Figure 6 – Spermine but not putrescine rescues methionine depletion-induced growth impairment. (a, b). Percentage of rings, trophozoites, immature and mature schizonts in *P. berghei* parasites growing in **(a)** MetS or MetD media and **(b)** IleS or IleD media supplemented with the polyamines SpM, Putr or both (Putr & SpM). **(c)** Parasitemia in synchronized *P. falciparum* 3D7 WT parasites cultured in MetD media supplemented with Putr, SpM or both. Parasitemia was measured by flow cytometry after SYBR Green staining. **a.** *n*=3 independent experiments, except for Putr + SpM (*n*=2) or **b.** *n*=2 independent experiments**. a, b.** Bars represent the mean percentage of parasites at each developmental stage with individual data points representing an independent experiment. Error bars represent the SD. **c.** Data represents the mean percentage of iRBCs ± SEM in *n*=2 independent experiments, each performed at least in triplicate.

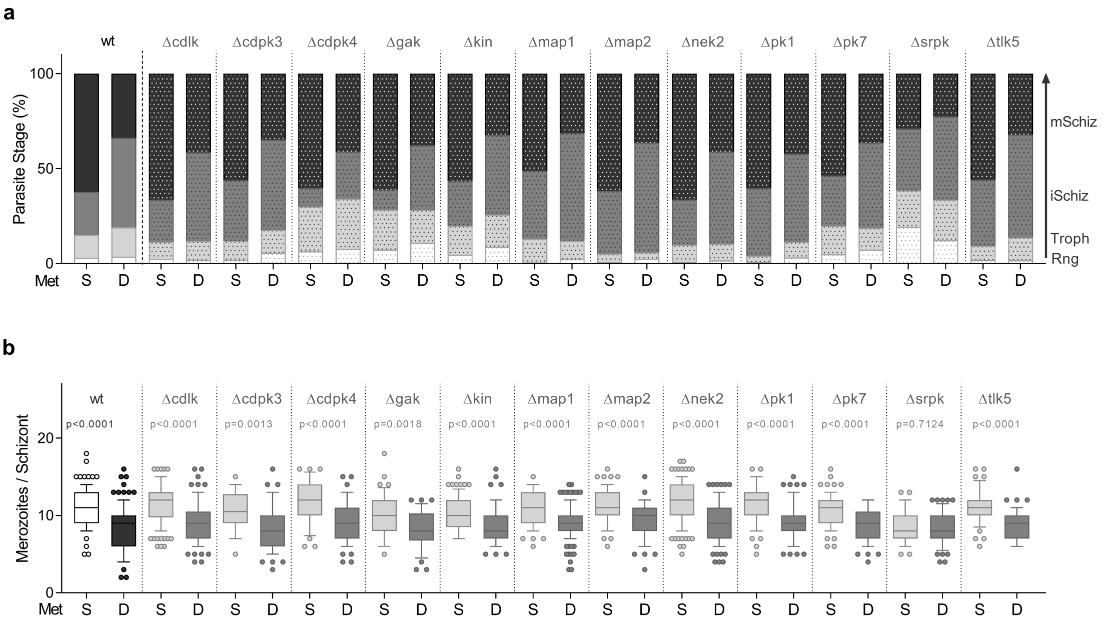

Supplementary Figure 7 – Phenotypic screen of *P. berghei* kinase knockout mutant parasites cultured *ex vivo* in AA deficient media. (a, b) Quantification of **(a)** intra-erythrocytic developmental stages and **(b)** mean merozoite numbers in *P. berghei* wild-type and kinase knockout mutant parasite lines using the *ex vivo* maturation assay. **a.** Parasite staging was performed in triplicate, averaged and repeated in n=2 independent experiments. Data is shown as mean percentage of parasites at each developmental stage. Individual data points are represented in **Supplementary Figure 8**. Rng: ring; troph: trophozoite; iSchiz: immature schizont; mSchiz: mature schizont. **b.** Data is represented as box-whisker plot (10-90 percentile) of mean merozoite number per schizont ± SD (2-way ANOVA). Boxplots show the data of n= 2 independent experiments and for the following number of schizonts (N): *P .berghei* wt: MetS, N=92; MetD, N= 99; Δcdlk: MetS, N =110; MetD, N=81; Δcdpk3: MetS, N= 28; MetD, N= 55; Δcdpk4: MetS, N= 33; MetD, N= 56; Δgak: MetS, N= 43; MetD, N= 34; Δkin: MetS, N= 85; MetD, N= 80; Δmap1: MetS, N= 82; MetD, N= 133; Δmap2: MetS, N= 64; MetD, N= 58; Δnek2: MetS, N= 112; MetD, N= 96; Δpk1: MetS, N= 49; MetD, N= 64; Δpk7: MetS, N= 69; MetD, N= 73; Δsrpk: MetS, N= 37; MetD, N= 54; Δtlk5: MetS, N= 44; MetD, N= 51.

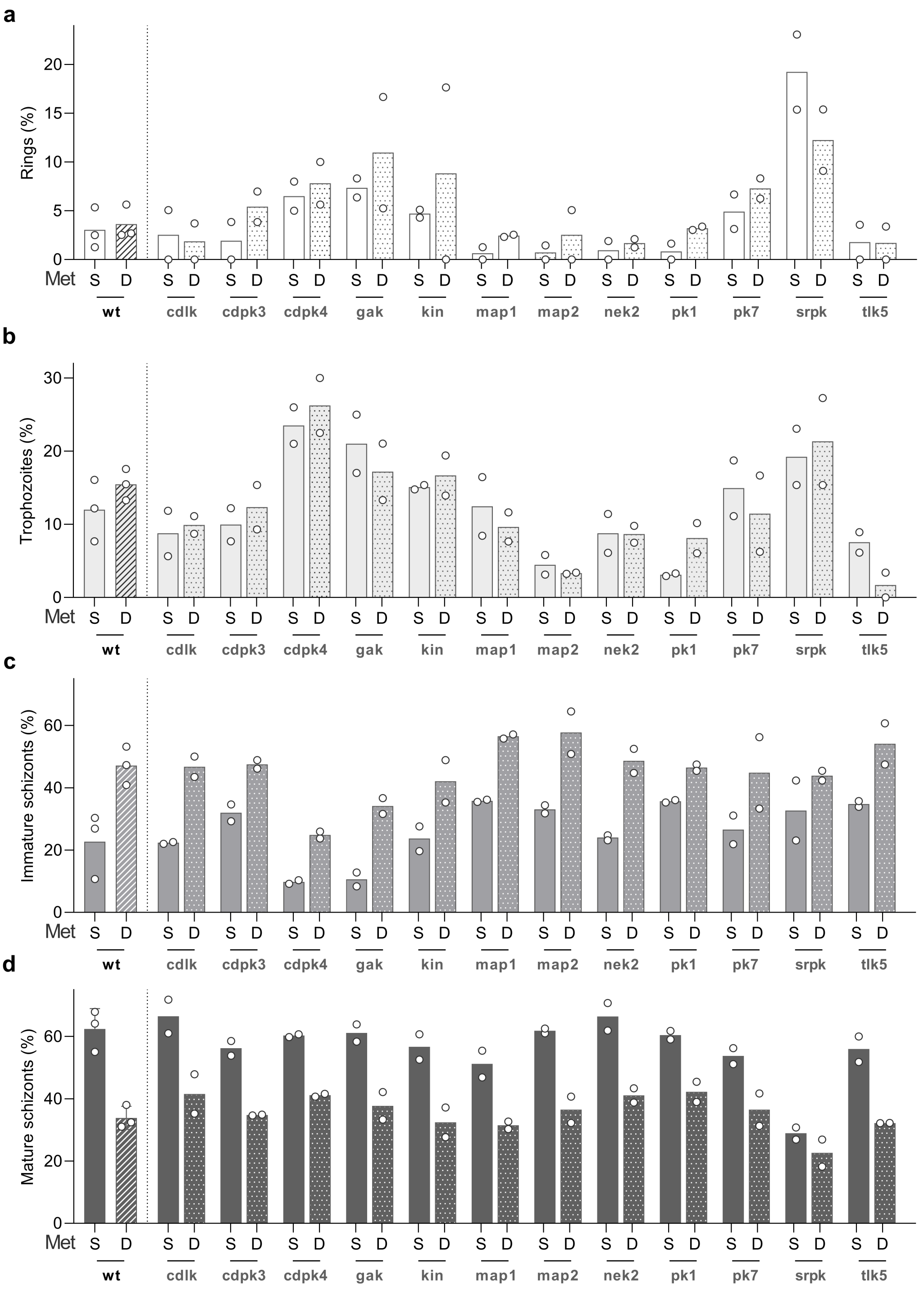

Supplementary Figure 8 – Parasite stage progression in wt and kinase mutant knockout lines culture in MetD media, using the *ex vivo* maturation assay. (a,d) Percentage of **(a)** ring-, **(b)** trophozoite-, **(c)** immature schizont- and **(d)** mature schizont-stage parasites cultured in MetS or MetD media. Bars represent the mean percentage of parasites at each developmental stage with individual data points representing an independent experiment. *n*=2 independent experiments.

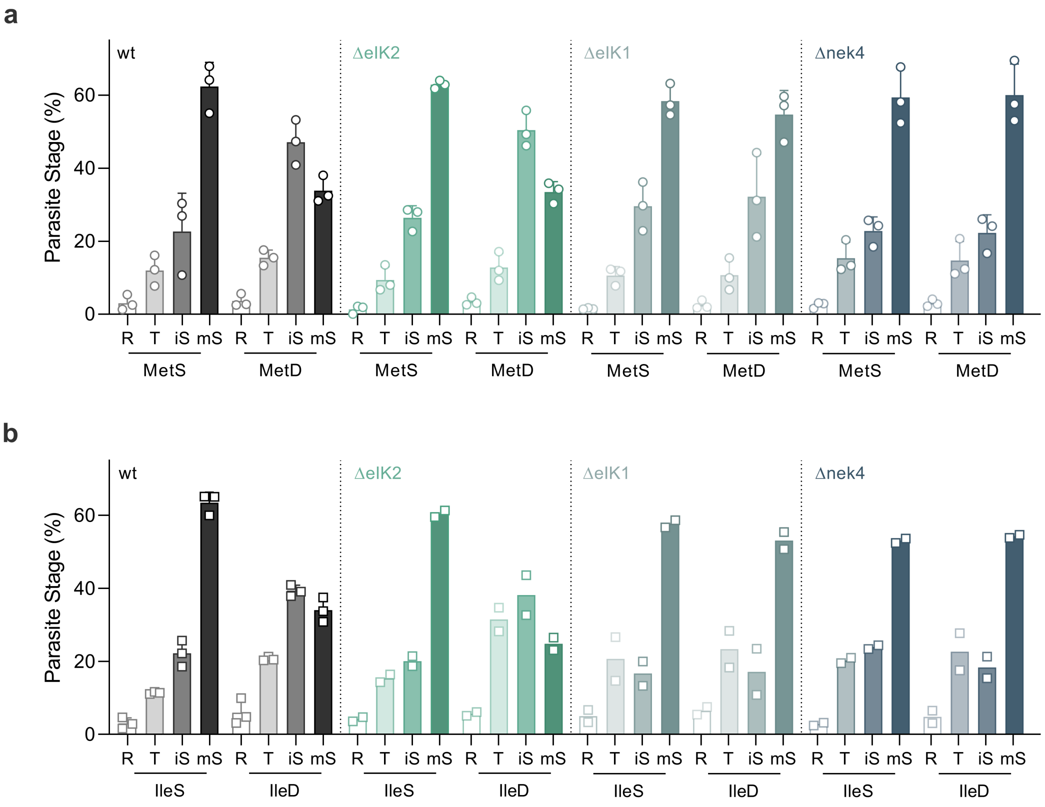

Supplementary Figure 9 – eIK1 and nek4, but not eIK2, regulate parasite development under AA deficiency. (a,b) Percentage of ring-, trophozoite-, immature schizont- and mature schizont-stage parasites cultured in **(a)** MetS or MetD media and **(b)** IleS or IleD media. Bars represent the mean percentage of parasites at each developmental stage with individual data points representing an independent experiment. *n*=3 independent experiments, except for kinase knockout mutants growing under IleS or IleD conditions (*n*=2).

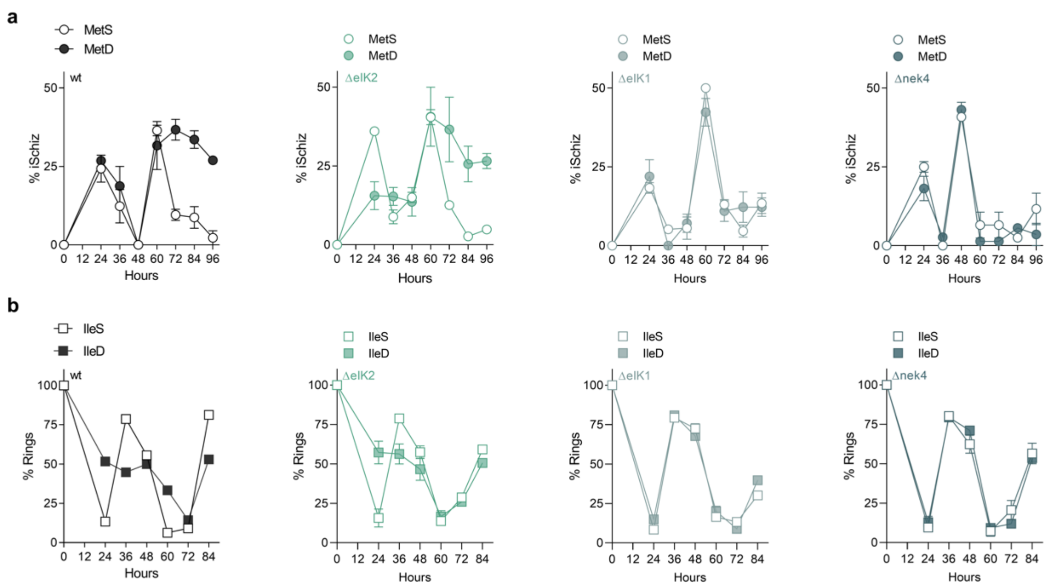

Supplementary Figure 10 – Impact of AA depletion in *P. falciparum* kinase knockout mutant parasites. **(a)** Time course analysis of immature schizont-stage *P. falciparum* 3D7 WT and kinase mutant parasites cultured in MetS or MetD media. (**b**) Time course analysis of ring-stage *P. falciparum* 3D7 WT and kinase mutant parasites cultured in IleS or IleD media. **a, b.** Data represents the mean percentage of **a.** immature schizonts or **b.** ring-stages ± SEM, each performed in triplicate, averaged and determined in n=2 independent experiments**.**

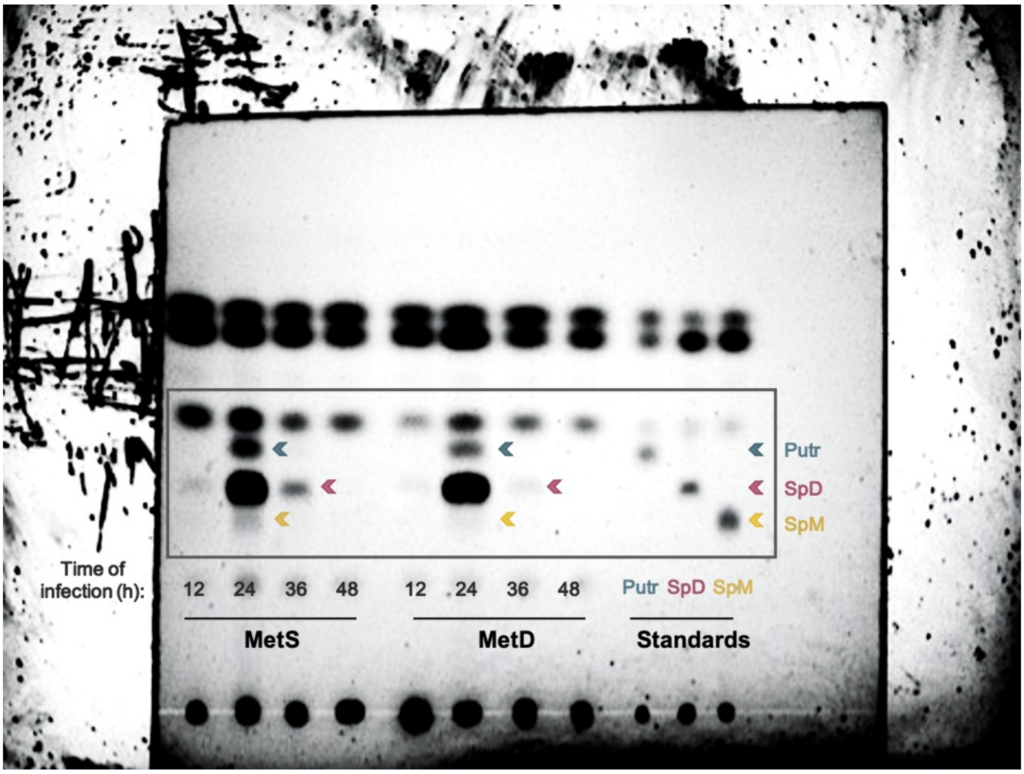

Supplementary Figure 11 - Raw Data of Figure 2f. The box shows where the image was cropped to generate the main figure.

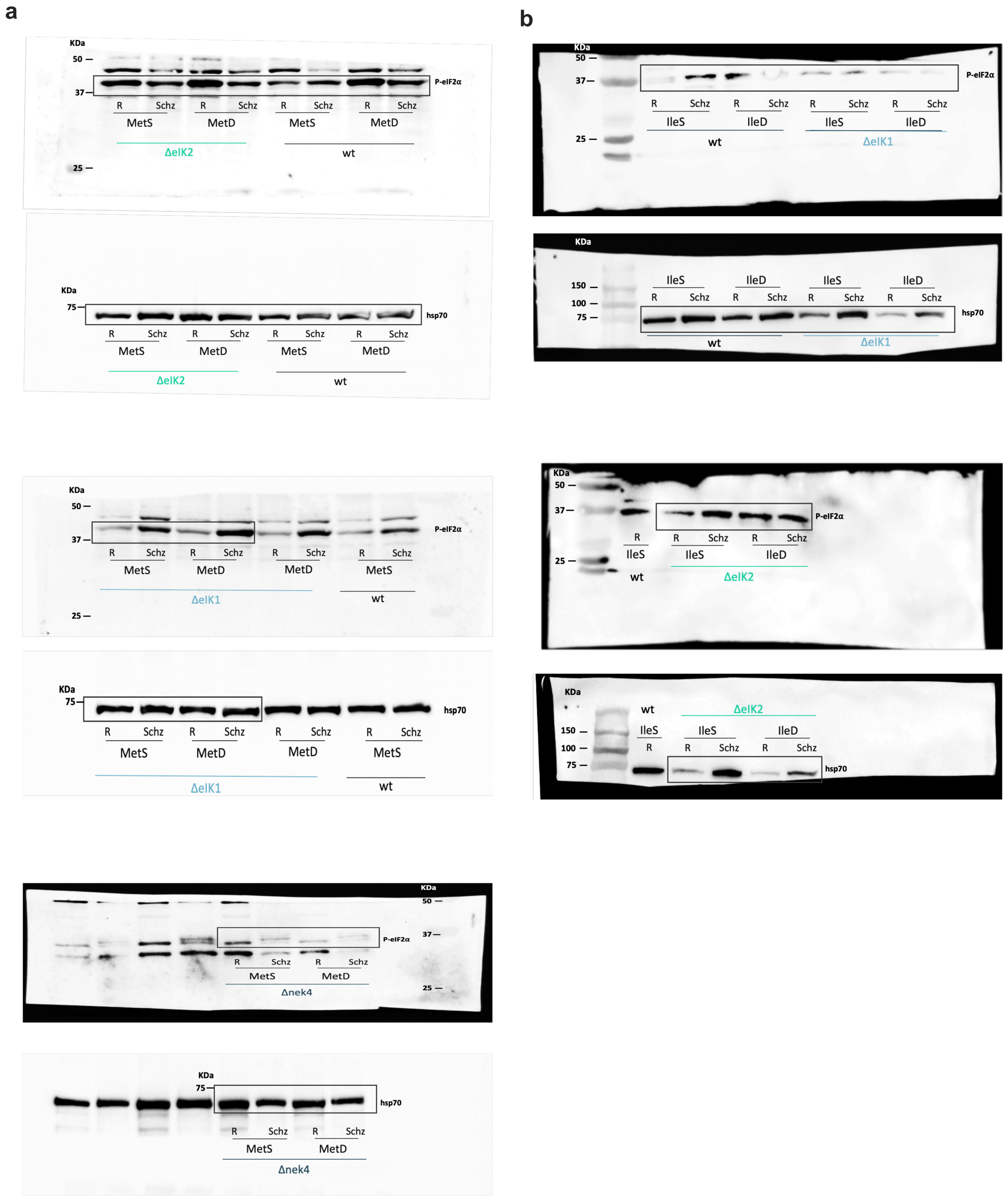

Supplementary Figure 12 - Raw Data of Figure 5. The boxes show where the images were cropped to generate the main figure.

PCR reaction	Primer pairs (Forward; Reverse)
***Pb*SAMS-HA-DD wt locus**	p1: 5’ TAGGTACCGAGGAAATTTTCTATTTACTTCG 3’<br>p3: 3’ ATGCGGCCGCCAACTAATAAAATCCAGGAAATA 5’
***Pb*SAMS-HA-DD modified locus**	p1: 5’ TAGGTACCGAGGAAATTTTCTATTTACTTCG 3’<br>p4: 3’ ATGCGGCCGCTCATCGCCGCTCCAGAATCTC 5’
**DNA quality control**	p1: 5’ TAGGTACCGAGGAAATTTTCTATTTACTTCG 3’<br>p2: 3’ TAGGGCCCATTTTTTAAAACATTTTTTTCGTG 5’

Supplementary Table 1 - Primers used for genotyping the *Pb*SAMS-HA-DD transgenic line.
