# Supplementary material for: A non-canonical sensing pathway mediates Plasmodium adaptation to amino acid deficiency
Source: Commun Biol. 2023 Feb 21;6:205. doi: 10.1038/s42003-023-04566-y (PMC9942083; doi:10.1038/s42003-023-04566-y)
Supplement: Supplementary file 3 — Description of Additional Supplementary Files [file 42003_2023_4566_MOESM3_ESM.pdf]

Description of Additional Supplementary Files

File name: Supplementary Data 1

Description: The Excel spreadsheet contains the raw data for all graphs, including main and supplementary figures.
