# Supplementary material for: A non-canonical sensing pathway mediates Plasmodium adaptation to amino acid deficiency
Source: Commun Biol. 2023 Feb 21;6:205. doi: 10.1038/s42003-023-04566-y (PMC9942083; doi:10.1038/s42003-023-04566-y)
Supplement: Supplementary file 5 — Reporting Summary [file 42003_2023_4566_MOESM5_ESM.pdf]

nature portfolio

Corresponding author(s): Maria M Mota

Last updated by author(s): Jan 18, 2023

Reporting Summary

Nature Portfolio wishes to improve the reproducibility of the work that we publish. This form provides structure for consistency and transparency in reporting. For further information on Nature Portfolio policies, see our Editorial Policies and the Editorial Policy Checklist.

Statistics

For all statistical analyses, confirm that the following items are present in the figure legend, table legend, main text, or Methods section.

n/a	Confirmed	
☐	☒	The exact sample size (*n*) for each experimental group/condition, given as a discrete number and unit of measurement
☐	☒	A statement on whether measurements were taken from distinct samples or whether the same sample was measured repeatedly
☐	☒	The statistical test(s) used AND whether they are one- or two-sided<br>*Only common tests should be described solely by name; describe more complex techniques in the Methods section.*
☒	☐	A description of all covariates tested
☒	☐	A description of any assumptions or corrections, such as tests of normality and adjustment for multiple comparisons
☐	☒	A full description of the statistical parameters including central tendency (e.g. means) or other basic estimates (e.g. regression coefficient) AND variation (e.g. standard deviation) or associated estimates of uncertainty (e.g. confidence intervals)
☐	☒	For null hypothesis testing, the test statistic (e.g. *F*, *t*, *r*) with confidence intervals, effect sizes, degrees of freedom and *P* value noted<br>*Give P values as exact values whenever suitable.*
☒	☐	For Bayesian analysis, information on the choice of priors and Markov chain Monte Carlo settings
☒	☐	For hierarchical and complex designs, identification of the appropriate level for tests and full reporting of outcomes
☒	☐	Estimates of effect sizes (e.g. Cohen's *d*, Pearson's *r*), indicating how they were calculated

Our web collection on statistics for biologists contains articles on many of the points above.

Software and code

Policy information about availability of computer code

Data collection	To acquire microscopy data, Leica Application Suite (LAS) and ZEN 2 (Blue version) were used.<br>To acquire Flow Cytometry data, BD FACSDiva™ Software (v6.2) was used.<br>To acquire WB images, ImageLab (v6.1.0) was used.
Data analysis	For image analysis, Fiji (version 2.1.0) was used.<br>For flow cytometry analysis, FlowJo X (v10.7.1) was used.<br>For statistical analysis, GraphPad Prism software (version 8.4.3) was used.<br>For data presentation, Adobe Illustrator (version CS4) was used.

For manuscripts utilizing custom algorithms or software that are central to the research but not yet described in published literature, software must be made available to editors and reviewers. We strongly encourage code deposition in a community repository (e.g. GitHub). See the Nature Portfolio guidelines for submitting code & software for further information.

Data

Policy information about availability of data

All manuscripts must include a data availability statement. This statement should provide the following information, where applicable:

- Accession codes, unique identifiers, or web links for publicly available datasets
- A description of any restrictions on data availability
- For clinical datasets or third party data, please ensure that the statement adheres to our policy

All data generated or analyzed during this study are included in this manuscript and its Supplementary Information. Source data underlying the graphs and charts presented in the main figures are available as Supplementary Data 1. Full blots are shown in Supplementary Information.

Human research participants

Policy information about studies involving human research participants and Sex and Gender in Research.

Reporting on sex and gender	N/A
Population characteristics	N/A
Recruitment	N/A
Ethics oversight	N/A

Note that full information on the approval of the study protocol must also be provided in the manuscript.

Field-specific reporting

Please select the one below that is the best fit for your research. If you are not sure, read the appropriate sections before making your selection.

☒ Life sciences ☐ Behavioural & social sciences ☐ Ecological, evolutionary & environmental sciences

For a reference copy of the document with all sections, see nature.com/documents/nr-reporting-summary-flat.pdf

Life sciences study design

All studies must disclose on these points even when the disclosure is negative.

Sample size	No statistical methods were used to determine sample size. Sample size is always specified in each figure legend and most experiments were performed with 3 independent replicates. Samples sizes are consistent with similar published results.
Data exclusions	For experiments using mice models, occasionally, animals were excluded from experimental groups due to inefficient infection (10-fold lower percentage of iRBCs than the group's average on the first day of parasitemia measurement) as assessed by FACS or microscopy. No other data were excluded.
Replication	All attempts of replication were successful. The number of replicates for all experiments is described in respective figure legends. Observed variation has been reported and contributes to the statistical analysis.
Randomization	Animals were randomly assigned to the different experimental groups. Microscopy analysis was performed in randomly acquired images.
Blinding	Authors were not blinded to the group allocation. However, data collection was performed in an unbiased manner and the analysis was performed on quantitative endpoints that are not subject to investigator bias.

Reporting for specific materials, systems and methods

We require information from authors about some types of materials, experimental systems and methods used in many studies. Here, indicate whether each material, system or method listed is relevant to your study. If you are not sure if a list item applies to your research, read the appropriate section before selecting a response.

Materials & experimental systems

n/a	Involved in the study
☐	☒ Antibodies
☐	☒ Eukaryotic cell lines
☒	☐ Palaeontology and archaeology
☐	☒ Animals and other organisms
☒	☐ Clinical data
☒	☐ Dual use research of concern

Methods

n/a	Involved in the study
☒	☐ ChIP-seq
☐	☒ Flow cytometry
☒	☐ MRI-based neuroimaging

Antibodies

Antibodies used	All antibodies used in this study are detailed in the Methods sections, in the "Immunobloting Analysis"and "Immunofluorescence Assay" subsections:<br>-rabbit α HA (clone C29F4, Cell Signaling Technology, Danvers, MA, USA)<br>-rabbit α phospho-eIF2α (S51) (119A11, Cell Signaling Technology)<br>-mouse α Pbhsp70 (produced in house)<br>-goat α mouse IgG, HRP conjugate (BML-SA204-0100, Enzo Life Sciences, Lausen, Switzerland)<br>-goat α rabbit IgG, HRP-linked Antibody (7074, Cell Signaling Technology)<br>-Alexa-488 conjugated donkey α mouse GFP (A21311, Invitrogen, Thermo Fischer Scientific)<br>-Alexa-647 conjugated donkey α rabbit IgG (A32795, Invitrogen, Thermo Fischer Scientific)<br>-DAPI (D1306, Invitrogen, Thermo Fischer Scientific
Validation	Validation statement of each antibody can be found on the manufacturer`s website, except for hsp70 which was produced in house and previously validated.<br><br>-HA: https://www.cellsignal.com/products/primary-antibodies/ha-tag-c29f4-rabbit-mab/3724<br>-phospho-eIF2α: https://www.cellsignal.com/products/primary-antibodies/phospho-eif2a-ser51-119a11-rabbit-mab/3597<br>-hsp70: (Tsuji M, et al., Parasitol Res. 1994)<br>-goat α mouse IgG, HRP conjugate: https://www.enzolifesciences.com/BML-SA204/goat-anti-mouse-igg-hrp-conjugate/<br>-goat α rabbit IgG, HRP-linked Antibody: https://www.cellsignal.com/products/secondary-antibodies/anti-rabbit-igg-hrp-linked--antibody/7074<br>-Alexa-488 conjugated donkey α mouse GFP: https://www.thermofisher.com/antibody/product/GFP-Antibody-Polyclonal/A-21311<br>-Alexa-647 conjugated donkey α rabbit IgG: https://www.fishersci.com/shop/products/igg-h-l-highly-cross-adsorbed-donkey-anti-rabbit-alexa-fluor-plus-647-polyclonal-secondary-antibody-invitrogen/PIA32795

Eukaryotic cell lines

Policy information about cell lines and Sex and Gender in Research

Cell line source(s)	P. falciparum NF54 and P. falciparum 3D7 were obtained through MR4 (www.mr4.org); P. falciparum sams-glmS transgenic parasite line was kindly provided by Björn Kafsack (Weill Cornell Medical College, New York, USA) and the P. falciparum 3D7 kinase deficient parasite lines were kindly provided by Mathieu Brochet (University of Geneva, Geneva, Switzerland) and Christian Doerig (Monash Biomedicine Discovery Institute, Clayton, Australia).
Authentication	None of the cell lines were authenticated in our lab.
Mycoplasma contamination	The cell lines were not tested for mycoplasma contamination.
Commonly misidentified lines<br>(See ICLAC register)	No commonly misidentified lines were used in this study.

Animals and other research organisms

Policy information about studies involving animals; ARRIVE guidelines recommended for reporting animal research, and Sex and Gender in Research

Laboratory animals	For this manuscript Balb/c mice were used, purchased from Charles River Laboratories. All mice used were males, 6-8 weeks of age.
Wild animals	No wild animals were used in this study.
Reporting on sex	Only male mice were used in this study
Field-collected samples	The study did not involve samples collected from the field.
Ethics oversight	All in vivo experiments were approved by the ORBEA committee of the iMM JLA and were performed according to national and European regulations.

Note that full information on the approval of the study protocol must also be provided in the manuscript.

Flow Cytometry

Plots

Confirm that:

☒ The axis labels state the marker and fluorochrome used (e.g. CD4-FITC).

☒ The axis scales are clearly visible. Include numbers along axes only for bottom left plot of group (a 'group' is an analysis of identical markers).

☒ All plots are contour plots with outliers or pseudocolor plots.

☒ A numerical value for number of cells or percentage (with statistics) is provided.

Methodology

Sample preparation	To assess parasitemia in the human malaria species, P. falciparum, the fluorescent dye, SYBR Green I (supplied as a 10,000x concentrate in dimethylsulfoxide, DMSO), was diluted in water to 1:1,000 ratio as a working concentration. 50 µL of P. falciparum cultured samples (2% haematocrit) were mixed with the same volume of SYBR Green I solution, incubated in the dark for 20min at room temperature and washed with 500µL PBS. Samples were acquired on a BD LSR Fortessa using the BD FACSDiva™ Software (v6.2). Data analysis was performed using FlowJo X software.<br>For the GFP-expressing rodent malaria species, P. berghei ANKA, parasitemia was assessed by flow cytometry analysis of one drop of tail blood collected in 200µL PBS.
Instrument	BD LSR Fortessa
Software	BD FACSDiva™ Software (v6.2)
Cell population abundance	For P. falciparum a total of 100,000-200,000 events was analyzed per condition.<br>For P. berghei ANKA a total number of 1 million events (day 1-2 of infection) or 100-200 thousand events (onwards) were acquired.<br>Percentage of iRBCs would range from 0 to ~15% depending on the day of infection.
Gating strategy	Gating strategy is described in Extended Data Figure 2. RBCs were selected on the basis of their size by gating first on FSC and SSC and, subsequently, on FITC/FL1 and PE/FL3 channels (to eliminate false positives associated to RBC auto-fluorescence). Within this population, iRBC were detected in the FITC/FL1 channel.

☒ Tick this box to confirm that a figure exemplifying the gating strategy is provided in the Supplementary Information.
